# Celebrating a year of collaboration: gratitude to our inaugural reviewers

**DOI:** 10.1093/ehjimp/qyae014

**Published:** 2024-03-01

As we embark upon a new year, it is with profound appreciation and a sense of accomplishment that I extend my heartfelt gratitude, on behalf of the editorial team and of the European Association of Cardiovascular Imaging (EACVI), to all the reviewers who played an integral role in shaping our inaugural year at the *European Heart Journal – Imaging Methods and Practice* (*EHJ-IMP*). Their dedication and expertise have been the bedrock of our endeavour to foster academic excellence.

In our inaugural year, our journal embarked on a journey to establish a platform for rigorous scientific inquiry and clinical exchange. The success of this endeavour is indebted to the reviewers who dedicated their time and expertise, transcending geographical barriers, to reach the aim of *EHJ-IMP*: ‘to image an open world, where experts share their latest results, knowledge and experience to improve patient care’.

The commitment demonstrated by our inaugural reviewers was instrumental in upholding the highest standards of quality and credibility. Their meticulous evaluations, insightful feedback, and constructive criticism have played a pivotal role in refining the articles that were published in the journal. Their contributions have enhanced the content of the journal and set a standard for the quality of scholarship we aim to uphold.

As we move forward, let’s keep fostering collaboration and excellence. With ongoing support from our reviewers, we’ll enhance scholarly discussions and make significant progress in advancing academic knowledge.

Dear reviewers, thank you for leading our first steps towards academic excellence.

Alberto Aimo

Stefano Albani

Ambreen Ali

Ana Almeida

Nicolas Amabile

Emmanuel Androulakis

Shehab Anwer

Andrea Baggiano

Andrea Barison

Georgios Benetos

Dominik Benz

Elisabetta Bianchini

Francesco Bianco

Sara Bombace

Ronny Büchel

Steele Butcher

Annagrazia Cecere

Rahul Choudhary

Giuseppe Ciliberti

Michele Coceani

Edoardo Conte

Marta Cvijic

Patrick Doeblin

Tiffany Dong Dong

Eoin Donnellan

Abdelrahman Elhakim

Francesco Faita

Federico Fortuni

Elena Galli

Pankaj Garg

Bernhard Gerber

Andreas Giannopoulos

Jan Gröschel

Marco Guglielmo

Krasimira Hristova

Ignatios Ikonomidis

Vikash Jaiswal

Alexandros Kasiakogias

Konstantinos Katogiannis

Simrat Kaur

Márton Kolossváry

William Kong

Olivier Lairez

Suvasini Lakshmanan

Virginie Le Rolle

Riccardo Liga

Alicia Maria Maceira González

Robert Manka

Stéphane Manzo-Silberman

Sarah Moharem

Antonella Moreo

Sara Moscatelli

Denisa Muraru

Laura Murphy

Shady Nakhla

Stefano Nistri

Mariaelena Occhipinti

Edyta Płońska-Gościniak

Giorgia Panichella

Elizabeth Paratz

Khrisha Patel

Amalia Peix

Martina Perazzolo Marra

Valeria Pergola

Rebecca Perry

Phillipe Pibarot

Tomaz Podlesnikar

Sandhir Prasad

David Prior

Francesca Pugliese

Nicola Riccardo Pugliese

Fabrizio Ricci

Andreas Rolf

Albert Roque

Alexia Rossi

Leyla Elif Sade

Anna Sannino

Ivan Stankovich

Ana Timoteo

Martin Ugander

Peter Van der Bijl

Tom Wang

George E Wesbey

Kyriakos Yiangou

Alessandro Zorzi

